# Social networks of American Indian youth on a Northern Plains reservation

**DOI:** 10.21307/connections-2019.046

**Published:** 2024-07-21

**Authors:** Katie Schultz, Jerreed Ivanich, Nancy Whitesell, Michael Siciliano, Tracy Zacher

**Affiliations:** 1 University of Michigan School of Social Work, 1080 S University Ave, Ann Arbor, MI 48109, USA; 2 Centers for American Indian and Alaska Native Health, Community and Behavioral Health, CU Anschutz, Nighthorse Campbell Native Health Building, 13055 East 17th Avenue, Aurora, CO 80045, USA; 3 Department of Public Policy, Management, and Analytics, University of Illinois at Chicago, 400 S. Peoria Street, Suite 2100, Chicago, IL 60607 USA; 4 Missouri Breaks Industries Research, Inc, 231 E. St. Joseph Street, Rapid City, SD 55701, USA

**Keywords:** Social network interventions, Substance use, Prevention, Native, Adolescents

## Abstract

Social relations and multigenerational networks remain a salient fixture of American Indian (AI) culture and survivance. Network data can describe the dynamic nature of social networks and the powerful role these relationships play in the development of behaviors in adolescence. Research in other populations has demonstrated how networks impact risk and resilience, but data on these factors are lacking among AI adolescents. There are reasons to expect that network structures may differ and that prevailing social network theories may not apply among this population. This paper describes ego and grade level networks of 9th and 10th grade AI youth (N = 263) in three diverse schools on a Northern Plains reservation. Aligned with prior research, we find that gender homophily plays a role in friendship formation. Unlike in other settings, race/ethnicity was not a significant predictor of friendship ties; this finding is not surprising given that 94% of the sample identified as being from this Northern Plains tribe. The descriptive findings also suggest that AI youth have a significant portion of family ties, even among their school-based networks. This may be a distinct feature of AI networks. Variation in networks across schools suggests unique community contexts that may make a universal approach to prevention development and implementation less effective. Within this tribal community, we find significant differences in the types, sizes, and potential mechanisms of tie formation. This underscores the importance of identifying network variations to implement targeted preventive interventions for feasibility, efficacy, and sustainability.

## Introduction

Innovative examinations of social networks among adolescents have proliferated in recent years (Ferguson et al., 2022; Hunter et al., 2019; Valente, 2023; Vassey et al., 2023), but such works have largely overlooked American Indian (AI) communities. Rigorous investigations of the social networks of AI youth, specifically reservation-based youth, are lacking, and there are reasons to expect that the developmental course and structure of social networks may differ in these contexts. Much of the social networks literature among non-AI youth points to the emerging influence of peer networks; parents tend to have a stronger effect on children’s attitudes and development before the transition to adolescence when peers become increasingly more relevant socializing influences (Hass et al., 2010). As adolescents grow in independence, they have increased maturity, autonomy, and expanding social environments that can facilitate stable and emotionally intimate friendships (Ferguson et al., 2022). The reliance on family, especially caregivers, as the primary source of behavior modeling and an informal social guidepost diminishes during this time (Hartup, 1993). Research has established that friendships during this period are affected by several factors, including popularity, social preference, age, and gender homophily (Currarini et al., 2010; Dwyer et al., 2010).

Many AI communities continue to be rooted in intricate communal relations. Cultural and traditional practices are often maintained through collateral kinship networks, and multigenerational networks (i.e., peer, family, kinship, and community) remain a core feature of AI culture and support in reservation communities. As in many communities, parents, grandparents, aunts, uncles, cousins, and other extended kin play strong social and supportive roles during adolescence. These social relations remain integral in AI communities that are often geographically isolated and, in many cases, under-resourced in terms of funding for health and educational services. While research in other communities suggests that reliance on family recedes during adolescence, that remains to be seen among AI populations. Many AI communities’ physical, spiritual, and economic wellbeing are intertwined with family and community relationships. Moreover, many AI societies are rooted in social structures in which family and community relationships define accepted duties and obligations to one another to ensure that communities thrive.

Several governing theories provide insight into potential factors that may contribute to network formation and maintenance (Monge & Contractor, 2003; Siciliano et al, 2021). These include homophily, preferential attachment, and reciprocity. Homophily is the principle that shared sociodemographic, behavioral, and intrapersonal characteristics lead to connections and that personal networks are generally homogeneous with regard to these characteristics (McPherson et al., 2001). Networks also tend to be centralized around a few highly popular actors. The tendency to want to form ties with already-popular actors, known as preferential attachment, can lead to networks that are highly centralized (Barabasi & Albert, 1999; Newman, 2001). Reciprocity measures the extent to which actions or social relationships directed from person A to person B are met with a mutual action or relationship from person B back to person A (Leider et al., 2009; Van Tilburg et al., 1991). Given the unique features of AI kinship and community formation and the special features of the reservation setting, one goal of this study is to determine how various governing network principles operate in reservation communities.

Social networks are critical in influencing youth behavior, and the knowledge of how networks operate can improve the design and success of efforts to prevent risky behaviors. Many structural factors related to historical trauma and systemic inequities drive disparities that are evident among AI adolescents (O’Connell et al., 2007; Whitesell et al., 2009, 2014). AI youth demonstrate early substance use initiation, which puts them at risk for subsequent problematic substance use rates and patterns (Nuño & Herrerra, 2022; Stanley et al., 2020; Whitbeck et al., 2014; Whitesell et al., 2014). These substance use patterns intersect with risks of suicide attempts or ideation (Barlow et al., 2012; Cole et al., 2020; Ivanich et al., 2021) and exposure to violence (Hautala & Sittner, 2021; Schultz et al., 2018). Progress in responding to and rectifying these inequitable risk factors could be bolstered by an expansion of prevention and intervention strategies drawing on naturally occurring and culturally relevant factors such as social networks, which may enhance effectiveness and sustainability.

Network interventions draw on social network data to identify and utilize social influence; create behavior change; or improve or achieve particular outcomes among individuals, organizations, or communities (Valente, 2012). In other populations, network interventions have been shown to be effective at improving a range of outcomes and behaviors, including smoking, bullying, and mental health (An, 2011; Hunter et al., 2019). Current applications of network interventions into AI communities are limited due to the lack of knowledge of the network characteristics among this population. Researchers working with AI and Alaska Native (AI/AN) populations have used social network data for novel assessments of intervention diffusion in community-based settings including on suicide prevention among AN adults (Wexler et al., 2019) and a family-based substance use intervention (Mason et al., 2023). However, this work remains limited. Moreover, advancements in prevention science utilizing social network approaches among AI adolescents require a better foundational understanding of their social network characteristics and how these may or may not vary within and across communities.

Limited research has described social networks among AI/AN adolescents. In one of the few studies with a reservation-based sample, researchers investigated social networks of AI young adults (*n* = 46; average age: 16) who had had a recent suicide attempt or ideation (Ivanich et al., 2022). Among this sample, the average network size was 9.57 (ranging from 0 to 23), with 5% of the network comprising cousins, and 35% of the alters in the network reported as Native (Ivanich et al., 2022). Another study described the social networks of younger AI youth (*n* = 256; average age: 9), who were part of a family violence-prevention program, 85% of whom lived on a reservation (Mason et al., 2023). Youth nominated on average five alters (could name up to nine people). Among these, 65% were listed as friends and 20% as cousins. Overall, 28% of the alters were family members with the majority reported as a peer-aged relative, and 73% reported as Native (Mason et al., 2023). In a study with Yup’ik adolescents (*n* = 47; average age: 15) in a rural AN community in southwest Alaska, the mean network size was six (Philip et al., 2016). In this study, youth were asked to nominate up to 13 alters who provided social support through love or discussing private matters. More adults were nominated (2.53) than youth (1.70), and the most common roles of the alters were parent (1.44), followed by sister/brother (1.30), and friend (1.09; Philip et al., 2016).

The National Longitudinal Survey of Adolescent Health (Add Health) data set is often referenced when considering adolescent social networks. In a systematic review of peer social network processes and adolescent health behaviors (Montgomery et al., 2020), the majority of the studies reviewed were from the United States (*n* = 40, 73%), and 70% of those (*n* = 28) used Add Health data—specifically data from between 1994 and 2002. The authors note that studies using Add Health data showed inconsistent findings and like other network studies can be difficult to compare given differences in sample sizes and methodologies. Moreover, they note that the studies using Add Health data are restricted to data from 1994 to 2002, and health behaviors among adolescents are likely different now, particularly with the growth of social media (Montgomery et al., 2020). In one study to report on AI adolescents (*n* = 316; average age: 15) using first wave Add Health data, the average network size across all groups was 4.99 (could nominate up to five male and five female friends) with AI youth showing higher levels of racial/ethnic heterogeneity compared to White and African American youth. The authors posit that this is likely because AI youth are typically in schools (with the exception of reservation schools or those in urban areas with dense AI populations) with fewer AI peers and so they have a greater range of ethnic/racial peers in their networks (Rees et al., 2014).

Findings reviewed here point to a scant and varied literature. The descriptive study presented here is part of a larger, mixed methods study that aimed to examine the extent to which existing social network theories and data metrics adequately characterize AI youth networks or how they may need to be expanded for this population. Schultz et al. (2023) describe the study protocol in more detail. This paper focuses on the first aim of that study, which was to describe the social networks of AI adolescents in this reservation setting and investigate differences and modifications required to accurately describe social networks among this population. To better understand the social networks of AI youth across diverse school contexts in one tribal community, this paper addresses two research questions: (1) What are the ego network characteristics of 9th and 10th grade AI youth in three schools on the reservation? and (2) How do grade-level networks vary across schools?

## Methods

### Community Setting

Northern Plains tribes have their own unique histories (e.g., migration, first contact with settlers, military resistance, food and subsistence, cultural and linguistic practices) as well as shared legacies of settler colonialism with other tribes—including the establishment of reservations, imposed governance structures, and boarding schools, to name a few. Northern Plains tribes occupy the northern half of the Great Plains, a sparsely populated region occupying approximately one-third of the continental United States. These tribes are an organization of what, at the beginning of the 18th century, were 30 distinct tribes. Today, the Great Plains region remains one of the most linguistically diverse regions for Native languages. Northern Plains tribes share a worldview of interconnectedness and oneness. One unifying aspect of this culture is an emphasis on the importance of social connections and relationships with defined duties and obligations to ensure community continuance and wellbeing.

As described previously (Schultz et al., 2023), this is a large reservation with several thousand residents living in small communities or in rural areas separated by many miles across the reservation. While significant structural and historical legacies compromise the health of some residents, strengths of the community, including kinship and supportive networks, are evident. The Nation is characterized by strong kinship structures that support the health of the community and should be integrated into effective preventive interventions to promote wellbeing among its citizens.

### Community Advisors

This study is guided by principles of community-based participatory research (Beals et al., 2003; Christopher et al., 2011; Wallerstein et al., 2018) and has benefitted from a long-standing partnership between a university center and this community. Prior to submitting an application for funding, Principal Investigators traveled to the reservation to meet with community members and schools to explore interest, value, and focus of the study. With funding, two review boards were established with individuals in communities where the participating schools were located. The Community Advisory Board included adults who work with youth and/or in prevention and intervention efforts related to youth substance use, suicidality, and exposure to violence. The Youth Advisory Board included two youth from each school (one female and one male) who were in the 11th or 12th grade. These board members were not eligible to participate in the study, but provided local knowledge of the schools and were close enough in age to the participants to provide meaningful feedback on the context and conduct of the study. Advisory Board meetings were held once a year, with additional consultation as needed. The Boards assisted in finalizing survey and interview questions and guiding study protocols, including recruitment and data collection.

### Sampling and Recruitment

The research team partnered with school leadership to recruit and collect social network surveys with 9th and 10th graders at three high schools on a Northern Plains reservation. This included signed statements of collaboration and financial compensation to the schools for the time and resources required to support recruitment and organize spaces for safe and secure data collection during school hours. Upon suggestion from the schools and with tribal approval, a passive parental permission process was utilized to reduce recruitment burden on the schools. Informational letters explaining passive consent and describing the study were sent home with students, handed out at school events, and emailed to parents and guardians. Students assented to participation before completing the survey. All protocols were reviewed and approved by the university and tribal research review boards.

All students enrolled in 9th and 10th grades at the participating schools were eligible, but not required, to participate in the study. The three participating schools represent various school types and community contexts: School 1—a small school in a geographically isolated village; School 2—a large tribal school in a larger village; and School 3—a private school with a college prep focus. We will refer to schools by their numbers in the sections below. All three schools include kindergarten through 12th grade (K-12). We had an overall response rate of 77% across the three schools. Specifically, 77% of the eligible students completed surveys in School 1 (*n* = 52), 76% in School 2 (*n* = 140), and 84% in School 3 (*n* = 71), for a total sample size of 263.

### Data Collection

Surveys were collected using Network Canvas, an interactive social network data-collection software (Birkett et al., 2021) implemented using tablet touch-based gestures. Adaptations for relevance to assessing network structures with AI adolescents included asking participants to nominate family and others outside of peer networks and asking students to identify family relationships within their peer networks. Moreover, we collected sociocentric (whole school networks) as well as egocentric networks to capture ties to family and the broader community. Student respondents, referred to as egos within social network literature, first completed a series of questions about themselves (demographic information and information about their own experiences with substance use, suicidal ideation, and exposure to violence). Next, they completed a social network assessment that consisted of three phases. *Phase 1*. Students were given name generator prompts for three distinct social groups in this order: (1) school-based friend, (2) family, and (3) nonschool/nonfamily connections. For the school-based networks, they were told, “Select up to 10 friends you are closest with in grades 7th–12th at your school.” Although we collected data from 9th and 10th graders, we expected that given the close-knit nature of the communities, their smaller population size, and extended kinship, students would hold friendships across a range of grades. School rosters were uploaded to assist in this step. As students began typing a name, matching names from the school roster would automatically populate. This improved the data quality by ensuring we knew which particular student was being nominated (e.g., if multiple students went by the same first name, we could differentiate based on full name). Students were then asked to nominate up to eight family members they were close with outside their school networks and then up to eight people they were close with outside of school or family networks. *Phase 2.* Students were asked a series of name interpreter questions for all people they identified in their networks, known as alters. These questions asked about attributes of the alters, including age, gender identity, socioeconomic status, race/ethnicity, type of relationship, length of the relationship, frequency of contact, and how close the ego felt to each alter. Among alters named as a school-based friend, we asked, “Are you related to this person?” and they could indicate by dragging and dropping the name of the alter into a yes or no category. For alters identified as family members, they were asked how they were related (e.g., parent/caregiver, grandparent, sibling, cousin, aunt/uncle). *Phase 3*. Finally, in order to assess network density and social embeddedness, students were provided an interactive visual display of their alters across all groups (school, family, and nonschool/nonfamily) and asked to indicate which alters were close to one another. Specifically, the question asked to indicate the “people who are close, even when you are not around.”

Data were collected at schools during class and lunch periods. We surveyed students late in the fall semester to allow networks to stabilize after the start of the school year. Students entered responses directly on tablets, with research team members available to answer or clarify questions. Youth received a $25 gift card for participating in the survey.

### Data Analysis

The survey produced network data that we classify into two types. The first type is ego network data. This data consists of all of the alters nominated by the ego across the three social groups (school, family, and nonschool/nonfamily). The second type is school-based network data. This data is a subset of the ego network data that focuses on the ties that exist among all of the 9th and 10th graders. Because each ego nominated friends in the 9th and 10th grade, we are able to create complete school networks for these grades.

Data analysis for this paper took place in three stages. First, demographic distributions were calculated for the study participants. This provides an overview of the sample for various characteristics valuable for understanding the contextual background of the respondents. Variables for this stage include age, gender, race/ethnicity, sexual orientation, current school, and length of attendance at current school.

Second, we analyzed the ego network data. We calculated several structural variables, including: (i) the average size of the ego network, (ii) the average proportion of school-based and family-based alters, (iii) the average proportion of nonschool, nonfamily-based alters, (iv) the average proportion of overall network that are family (combined peer related and family alters), (v) the number of Native alters, (vi) the average proportion of same-gender alters, (vii) the average proportion of alters who share the same socioeconomic status, and (viii) the average number of alters in different age categories. We compared these variables for significant differences across schools using one-way ANOVA tests with a Turkey Honest Significant Differences post-test.

**Figure 1:**
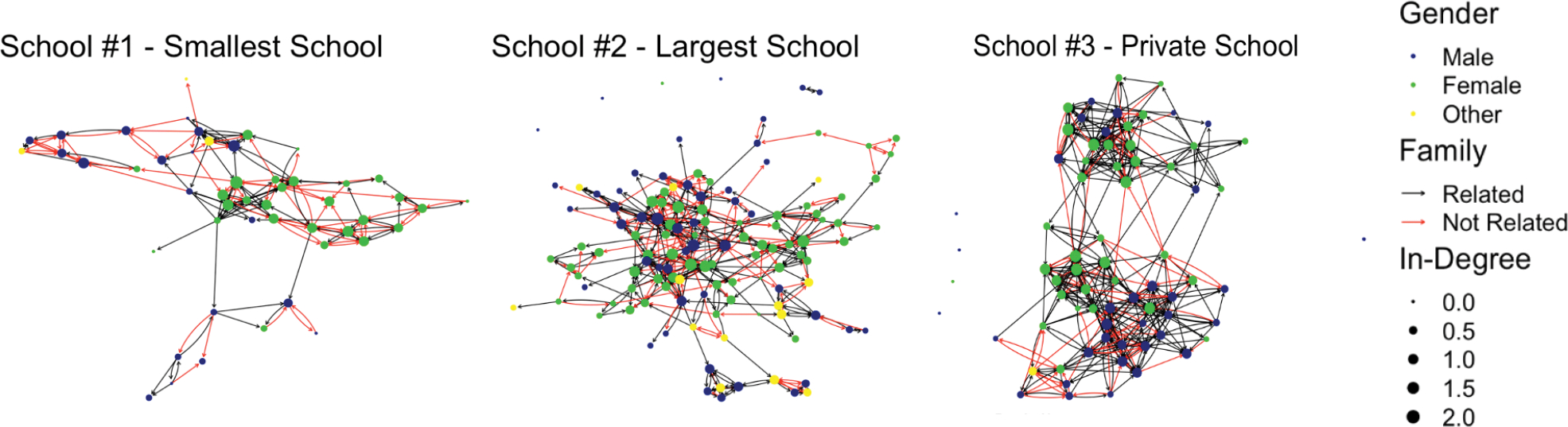
School networks, 9th and 10th grades, by family relation and gender.

**Figure 2:**
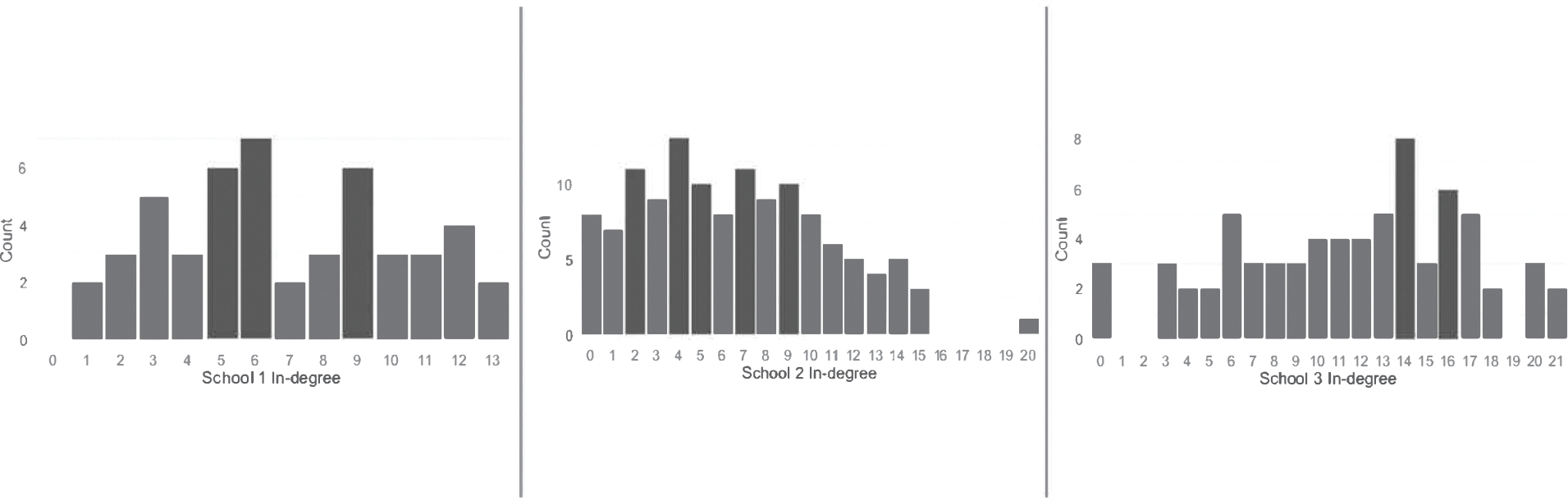
Histograms of In-degree distributions for each school site.

Lastly, we examine the school-based networks. These are the complete networks of 9th and 10th grades for each of the three schools. As noted above, these networks are constructed from the ties among the 9th and 10th graders. We provide information on the density, average in-degree (nominations of an individual from others in the network), centralization, the proportion of same-gender ties, and the proportion of family ties. We provide visualizations of the schools (Figure 1) and histograms of the in-degree distributions for each school (Figure 2). This initial description of these networks is limited to high-level descriptions of the overarching structures in the data; rigorous empirical data analyses of these structures and comparisons to networks in other populations are beyond the scope of this paper.

## Results

### Ego Demographics

The average age of the respondents was 15 years. The sample includes slightly more young men (51%) than women (41%) with 8% of the participants identifying as non-binary, trans, Two-Spirit, or another gender. The majority of the students (72%) identified their sexual orientation as straight. Just over half of the sample (53%) came from one school, the largest school in the study, and students reported having spent, on average, almost 4 years in their current school. The vast majority (94%) self-identified as a member of this Northern Plains Tribe alone or in combination with another race/ethnicity. See Table 1 for detailed demographics of the sample.

### Ego Networks

Ego networks represent all the people the youth nominated, including school, family, and nonschool and nonfamily alters. Table 2 details differences in ego networks across the three schools. The average size of ego networks was 14, meaning that participants typically nominated about 14 alters in their networks. Across schools, approximately half of students’ networks were made up of school-based alters (ranging from 45% to 52%) and about a third were comprised of family members outside of school (29%–33%). When we combine the school-based alters that participants reported being related to with family alters, we find that about half (45%–55%) of the overall networks are made up of family members. Not surprisingly, alters ranging in age from 13 to 18 made up the largest proportion of students’ networks. The majority of alters nominated within the networks were of the same gender as the participant, suggesting gender homophily.

When examining for statistically significant differences between schools, we found School 3 to be unique in some respects compared to the other two schools. For example, although School 3 was not the largest school, the ego networks of students in the school were slightly larger than the other two schools. Specifically, youth in School 3 nominated, on average, about three more alters in their networks than did youth in the other two schools. Respondents from School 3 also had a slightly lower proportion of their network that was the same gender and more alters in the 13- to 15-year-age range. School 3 also had a larger number of alters in the 31- to 60-year-age range compared to School 1 and more alters in the over 61 age range compared to School 2. We did not find any significant differences in the ego network characteristics between students from Schools 1 and 2.

### School Networks

For the school networks, we subset the ego network data to just school-based alters from 9th and 10th grades. We did this because for these grades, we had complete network data on the ties among the students who responded to the survey. Figure 1 shows networks by relationships and gender across the three school contexts. The node (circles in the image representing individual students) color reflects gender (green indicates male; blue female; yellow another gender identity). Red edges (lines connecting nodes) represent family relations; black represents nonfamily. Examining the color of those lines, we see examples of instances when one individual identified the other as family while the other said they were not related. Across all three schools, there were disagreements on family relation status in 16% of the school-based ties (School 1%–14%, School 2%–22%, School 3%–12%).

Table 3 provides descriptive statistics for the complete networks of 9th and 10th graders in each of the three schools. Because the network itself is the unit of analysis, and we are examining just three schools, there are no tests for statistical differences for these measures. Schools 1 and 3 showed greater density than School 2. Density is a measure of the overall level of connectedness and is calculated as the ratio of existing ties to total possible ties in a network. In other words, it is the proportion of ties that are present. The observed differences in density among the schools are driven, in part, by differences in the overall network size. We also calculated the average in-degree in each school, which is less influenced by the overall size of the network. In-degree indicates the number of times an individual was nominated by another in the network. Similar in-degrees were found across Schools 1 and 2 (3.4 and 3.3, respectively), while School 3’s indegree was over two degrees greater at 5.7. As given in Figure 1, where we see some clustering of nodes by gender, there is a strong tendency toward homophily based on gender, with 67%–79% of all ties in the networks being between students of the same gender. The percentage of family members in these school networks ranges from 24% to 46%. In School 1, for example, youth indicated that they were related to 46% of the alters they nominated in 9th and 10th grades.

## Discussion and Implications

To our knowledge, this is one of the first studies to collect ego and complete grade level social network data among this population. Descriptions of network characteristics are necessary to build a body of evidence that is foundational for the use of promising prevention approaches offered by social network science with AI/AN adolescents and in other communities with similar social and community contexts. Notably, our study offers three crucial findings for the use of social network interventions in AI/AN communities and others with similar attributes: (1) the most common approach to network interventions (key opinion leaders) may not be appropriate for use in this population; (2) family relations may serve a particularly vital role in the development and influence of networks in this population; and (3) multilevel interventions, integrating individual and community network influences, may be required.

Aligned with prior research in non-AI settings (Goodreau et al., 2009; Shrum et al., 1988) and with some limited work with AI youth (Edwards et al., 2022), we find that gender homophily plays an important role in friendship formation. Unlike other settings, race/ethnicity was not a significant predictor of friendship ties; not surprising given that 94% of the sample identified as being from this Northern Plains tribe. The descriptive findings also suggest that AI youth have a significant portion of family ties, even among their school-based networks. This may be a distinct feature of AI networks. However, much of the published literature is peer-based and does not include family ties among peer networks. It is hard to conclude if it is the case that AI populations have more family ties in their networks or that this has not been explored in previous works. Notably, the school networks are limited to reciprocated relationships within 9th and 10th grades, so this is likely a conservative estimate of school-based family ties.

The high proportion of family members may also be some function of the rurality, small populations, or low mobility in these community settings. In fact, we did find the largest percentage of family members in school-based networks in the smallest, most rural school. Few studies have explicitly focused on social networks of adolescents in rural communities within the United States. Researchers examining social support among 9th–12th graders (94% White; *n* = 600) in three rural counties in southwest Virginia found that when asked to name adults important to them, the average network size was 7.51 (students could nominate up to 10 people) with an average of 6 family members in the network (Singh & Dika, 2003). This lends some credit to considering how much are the family proportions of social networks a function of cultural or kinship features, or are they more related to rurality among this population. Knowing more about the influence of the rural nature of the setting for a variety of populations could be beneficial in understanding how they may differ from more commonly studied urban populations. This could benefit other historically neglected communities in utilizing the promise of social network science.

Visual patterns in the social networks (Figure 1) suggest that ways of defining family relations varied across students. We are not sure if this is a result of issues of measurement or broader, perhaps more inclusive conceptualizations of family relations in this population. For example, the way that the question is asked (“Are you related to this person?”) may be too broadly worded in a community in which being a relative has wide-ranging and potentially multiple ways of defining relationships including biological families as well as extended kinship networks. In some AI communities, there is no differentiation between nuclear and extended family. Further exploration of this should be done with community consultation, qualitative inquiry, and/or exploring different ways of asking about family relationships in survey questions to refine the measurement of family networks with AI populations. This may be a valuable contribution for researchers and community members doing social network analyses in other communities that have similarly complex extended kinship features.

Another key feature of our findings demonstrates that even within this one tribal community, we find significant differences in the types, sizes, and potential mechanisms of social tie formation. This is a small sample in one reservation, with one tribe, limited to two grades in three schools and the fact that we still see variation among the schools is compelling. This work shows the importance of understanding networks that are rooted in place and in microcultures even within larger community boundaries such as a reservation. It rejects the common treatment of Native cultures and peoples as monolithic. Furthermore, it suggests that getting this kind of data within individual schools can help identify specific strategies for intervention and prevention to maximize feasibility, effectiveness, and sustainability.

The developmental literature highlights the role school transitions (elementary to middle school and middle to high school) play in adolescent networks, but K-8 schools are common in this community; youth who attend K-8 schools typically transition into K-12 schools for high school. This is in contrast to the feeder school systems typical of urban areas in which youth transition schools at 6th and 9th grades and this may influence the way networks are developed and sustained across early adolescence. Longitudinal data should be collected to study the developmental course of AI youth networks over critical stages of adolescence within these specific school contexts to inform network-intervention strategies tailored for these youth.

### Implications for Preventive Interventions

The analysis of social networks offers promising avenues for developing and implementing targeted preventive interventions that may be more feasible and sustainable, particularly among under-resourced communities. The most deployed social network intervention focuses on key opinion leaders (An, 2011; Hunter et al., 2019). These individuals are optimally positioned in a network, typically based on their in-degree centrality, to use their status in the network to promote and legitimize behavioral change. An indicator of centrality includes in-degree, in this case, a measure of how many times an individual was nominated by someone in their school network as a friend. In-degree is often interpreted as representing a form of popularity or influence. Previous research using Add Health data has found evidence of preferential attachment and tendencies toward centralized networks (Schaefer & Simpkins, 2014). A relevant finding from our data was that the school-based networks in these schools were not highly centralized. This is evident in Figure 2, which shows the histograms of the in-degree distributions for each of the three schools. All of the schools follow a somewhat normal distribution, rather than a power-law distribution. Consequently, there may be no clear opinion leaders to target as change agents. This suggests that network interventions engaging key opinion leaders may not translate for this population. Thus, future program development should consider other network-intervention strategies. For instance, segmentation intervention strategies offer one possible approach where group detection algorithms are used to find cohesive subgroups in a network. Interventions are then simultaneously targeted to members of those groups. Segmentation strategies rely on group-based learning and peer support to reduce the risk associated with behavioral change (Valente, 2012).

This study focused on affective relationships, the type of relationship most common in the youth intervention and prevention literature. This does limit our ability to make claims about the patterns of other important relations. For example, networks may be highly centralized when looking at advice relationships. Future work might consider examining multiple types of relationships in order to gain more insight into multiplexity and the factors at work in shaping tie decisions across a range of relation types.

Variation in networks across schools on this reservation suggests potentially unique community contexts that may make a universal approach to prevention development and implementation less effective. Our visual and descriptive data suggest variation worth further exploration. For example, we had anticipated a larger proportion of family members in the overall networks of youth at School 1, given its smaller size and more geographically isolated location. This is indicated in part by the larger proportion of family in their grade level networks but not across their whole ego networks when compared to proportions at other schools. However, nearly half (46%) of their grade level networks were family, and this may have implications for interventions. Future work might consider the multilevel diffusion of ideas or behaviors, integrating family and peer-based networks. School 3 showed some unique features not included in our data that may be a result of it being a private religious school with a college prep focus. In our review of the literature on the social networks of AI/AN youth, we found three published manuscripts. Without foundational work on network characteristics, we are limited in our ability to identify areas of adaptation in both measurement and the use of social networks in prevention efforts. It also limits our ability to identify intertribal variability. More research should be done to identify ways in which this work should be adapted and localized across tribal communities to enhance measurement and effectiveness of interventions.

Many preventive interventions remain focused on the individual (Paskett et al., 2016). Variation we observed in networks across schools suggests that we should consider further examination of school and community differences that could help explain how these contexts influence network formation and maintenance. In addition to refining questions asked about school networks, one possible way to do this would be to collect community-based networks involving school-based programs and other youth-serving agencies and services. Integrating data from AI youth social networks with data describing these community-level networks may help us better understand when, where, and how to intervene at individual and institutional levels. This is particularly relevant for communities operating with a scarcity of resources and likely to benefit from multilevel prevention approaches.

Overall, AI communities vary in both context (remote, rural, urban) and culture in ways that are likely to be related to the formation of social networks. Findings from this study are limited to three schools within one Northern Plains reservation community, which limits generalizability, although findings may be applicable to other Northern Plains tribes in the region. Nonetheless, this study represents an important step toward documenting the social network structures of AI youth, and findings and insights may be relevant to other communities with similar attributes including rurality or extended kinship networks.

## Figures and Tables

**Table 1.** Description of the sample (N = 263).

Statistic	N	Mean/%
Age (years)	263	15.38
Gender		
Female	107	41%
Male	133	51%
Non-binary, trans, two-spirit, another gender^a^	23	8%
Sexual orientation		
Straight	189	72%
Bisexual/pansexual/omnisexual	39	15%
Questioning	19	7%
Gay/lesbian	11	4%
Two-spirit or asexual^a^	5	2%
School		
School 1	52	20%
School 2	140	53%
School 3	71	27%
Number years at school	263	3.67
Race/ethnicity (self-identified)		
This Northern Plains tribe only	75	65%
This Northern Plains tribe in combination	170	29%
Other AI or AN	6	2%
Other race/ethnicity	37	14%

a Combined for privacy.

AI, American Indian; AN, Alaska Native.

**Table 2.** Ego networks by school.

	School 1				School 2				School 3				Sig < 0.05^a^		
Statistics	Mean	St. dev.	Min	Max	Mean	St. dev.	Min	Max	Mean	St. dev.	Min	Max	1 vs 2	2 vs 3	1 vs 3
Network size	13.10	7.85	1.00	26.00	13.14	7.82	1.00	26.00	15.91	6.15	2.00	26.00	-	^a^	^a^
Proportion same gender	0.76	0.18	0.14	1.00	0.75	0.21	0.09	1.00	0.65	0.20	0.05	1.00	-	^a^	^a^
Proportion same SES	0.32	0.33	0.00	1.00	0.29	0.34	0.00	1.00	0.33	0.33	0.00	1.00	-	-	-
Proportion related^b^	0.55	0.25	0.00	1.00	0.50	0.24	0.00	1.00	0.45	0.20	0.00	1.00	-	-	-
Number of native alters	11.43	7.03	0.00	26.00	11.86	7.74	0.00	26.00	14.97	5.80	2.00	26.00	-	-	^a^
Proportion alter type															
School alters	0.52	0.30	0.00	1.00	0.45	0.32	0.00	1.00	0.51	0.24	0.00	1.00	-	-	-
Family alters	0.29	0.23	0.00	1.00	0.33	0.25	0.00	1.00	0.33	0.19	0.00	1.00	-	-	-
Other alters	0.18	0.19	0.00	1.00	0.22	0.21	0.00	1.00	0.16	0.13	0.00	0.50	-	^a^	-
Age groups															
<10	0.18	0.49	0.00	2.00	0.23	0.91	0.00	9.00	0.21	0.45	0.00	2.00	-	-	-
10–12	0.29	0.65	0.00	3.00	0.36	0.86	0.00	6.00	0.20	0.53	0.00	2.00	-	-	-
13–15	4.29	4.36	0.00	18.00	4.74	4.30	0.00	16.00	7.56	4.55	0.00	17.00	-	^a^	^a^
16–18	4.18	4.38	0.00	18.00	3.46	3.63	0.00	20.00	3.01	3.32	0.00	18.00	-	-	-
19–30	1.43	1.41	0.00	5.00	1.43	1.94	0.00	9.00	1.39	1.76	0.00	10.00	-	-	-
31–60	1.61	1.69	0.00	8.00	1.98	2.25	0.00	10.00	2.59	2.04	0.00	8.00	-	-	^a^
61+	0.49	1.17	0.00	5.00	0.35	0.76	0.00	3.00	0.64	0.92	0.00	4.00	-	^a^	-

a Examines for statistical difference across the three schools (e.g., School 1 vs School 2).

b Includes friends they reported as being related to and family alters.

**Table 3.** School networks.

	Network size	Density	Average in-degree	Centralization	Proportion same gender ties	Proportion family ties
School 1–9th & 10th	49	0.071	3.4	0.097	0.79	0.46
School 2–9th & 10th	128	0.026	3.3	0.053	0.76	0.39
School 3–9th & 10th	70	0.083	5.7	0.092	0.67	0.24
